# Chromatin remodeling in replication‐uncoupled maintenance DNA methylation and chromosome stability: Insights from ICF syndrome studies

**DOI:** 10.1111/gtc.12850

**Published:** 2021-05-07

**Authors:** Motoko Unoki

**Affiliations:** ^1^ Division of Epigenomics and Development Medical Institute of Bioregulation Kyushu University Fukuoka Japan

**Keywords:** chromatin remodeling, chromosome stability, DNA methylation, DNA repair, DNA replication, homologous recombination, ICF syndrome, multiradial chromosome, nonhomologous end joining, pericentromere

## Abstract

Immunodeficiency, centromeric instability, and facial anomalies (ICF) syndrome is characterized by frequent appearance of multiradial chromosomes, which are distinctive chromosome fusions that occur at hypomethylated pericentromeric regions comprising repetitive sequences, in activated lymphocytes. The syndrome is caused by mutations in *DNMT3B*, *ZBTB24*, *CDCA7*, or *HELLS*. De novo DNA methylation is likely defective in patients with ICF syndrome harboring mutations in *DNMT3B*, whereas accumulating evidence suggests that replication‐uncoupled maintenance DNA methylation of late‐replicating regions is impaired in patients with ICF syndrome harboring mutations in *ZBTB24*, *CDCA7*, or *HELLS*. ZBTB24 is a transcriptional activator of *CDCA7*, and CDCA7 and HELLS compose a chromatin remodeling complex and are involved in the maintenance DNA methylation through an interaction with UHRF1 in a feed‐forward manner. Furthermore, our recent studies possibly provided the missing link between DNA hypomethylation and the formation of the abnormal chromosomes; it could occur via aberrant transcription from the hypomethylated regions, followed by pathological R‐loop formation. The homologous‐recombination dominant condition caused by a defect in nonhomologous end joining observed in several types of ICF syndrome could facilitate the formation of multiradial chromosomes. Here, the latest knowledge regarding maintenance DNA methylation and chromosome stability provided by those studies is reviewed.

## Graphical abstract

DNA methylation is maintained in either replication‐coupled or ‐uncoupled manners. The CDCA7/HELLS chromatin remodeling complex, which is mutated in ICF syndrome, contributes to centromeric/pericentromeric stability plausibly by facilitating the replication‐uncoupled process.

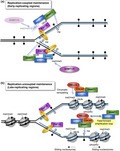

## 1 INTRODUCTION

DNA is wrapped around histone octamers to form nucleosomes, and nucleosomes compose chromatin. A lightly packed form of chromatin is called euchromatin, whereas a tightly packed form of chromatin is called heterochromatin. In mammals, heterochromatin is tightly associated with the di‐ and tri‐methylation of histone H3 lysine 9 (H3K9me2/me3) and of the C5 positions of cytosine in the CpG context (called DNA methylation hereafter). DNA methylation plays a major role in the transcriptional regulation of gene expression, silencing of transposable elements, maintenance of genome integrity, and chromosome stability, and is classified into two types (Unoki, 2019). One is de novo DNA methylation, in which methyl groups are added to cytosine residues in unmethylated DNA. DNMT3A and DNMT3B are responsible for this type of DNA methylation (Okano et al., 1999). The other is maintenance DNA methylation, in which methyl groups are added to unmethylated cytosine residues in hemi‐methylated DNA after DNA replication. DNMT1 and its cofactor, ubiquitin‐like containing plant homeodomain (PHD) and really interesting new gene (RING) finger domains 1 (UHRF1), are indispensable for this process (Sharif et al., 2007). In the last two decades, many additional factors have revealed to be involved in the maintenance DNA methylation process. Cell division cycle associated 7 (CDCA7) and helicase lymphoid specific (HELLS), which compose a chromatin remodeling complex possessing nucleosome‐sliding activity (Jenness et al., 2018), are two of such factors. Because both proteins are mutated in a subset of patients with immunodeficiency, centromeric instability, and facial anomalies (ICF) syndrome (Thijssen et al., 2015), who exhibit centromeric/pericentromeric DNA hypomethylation and chromosome instability, the insights from recent studies regarding these proteins highlight the importance of chromatin remodeling in DNA methylation at centromeric/pericentromeric regions for chromosome stability (Unoki et al., 2019, 2020). In this review article, the latest knowledge on maintenance DNA methylation is summarized, and the possible roles of ICF‐syndrome‐related proteins in maintenance DNA methylation and chromosome stability are discussed.

## 2 REPLICATION‐COUPLED AND REPLICATION‐UNCOUPLED MAINTENANCE DNA METHYLATION

During embryogenesis, DNA methylation patterns that are specific to each cell type are established by DNMT3A and DNMT3B. Subsequently, these patterns are maintained by the DNMT1/UHRF1 complex throughout the individual's lifetime (Unoki, 2019). UHRF1 (also known as Np95 and ICBP90) is a unique protein harboring a ubiquitin‐like (UBL) domain, a tandem Tudor domain (TTD), a PHD finger, a SET and RING‐associated (SRA) domain, and a RING domain. The TTD recognizes H3K9me2/me3 and di‐ and tri‐methylated DNA ligase 1 (LIG1K126me2/me3) (Ferry et al., 2017; Karagianni et al., 2008), the PHD finger recognizes the unmethylated N terminus of histone H3 and PCNA‐associated factor 15 (PAF15) (Arita et al., 2012; Nishiyama et al., 2020), the SRA domain recognizes hemi‐methylated DNA (Arita et al., 2008; Avvakumov et al., 2008; Hashimoto et al., 2008; Unoki et al., 2004), and the RING domain mono‐ubiquitylates multiple lysine residues of histone H3 in nucleosomes and those of PAF15 (Ishiyama et al., 2017; Karg et al., 2017; Nishiyama et al., 2013; Qin et al., 2015). The UBL domain facilitates the RING‐mediated ubiquitylation, the SRA‐mediated recognition of hemi‐methylated DNA, and chromatin association via interaction with HELLS (alternative name is LSH) (DaRosa et al., 2018; Foster et al., 2018; Han et al., 2020).

**FIGURE 1:**
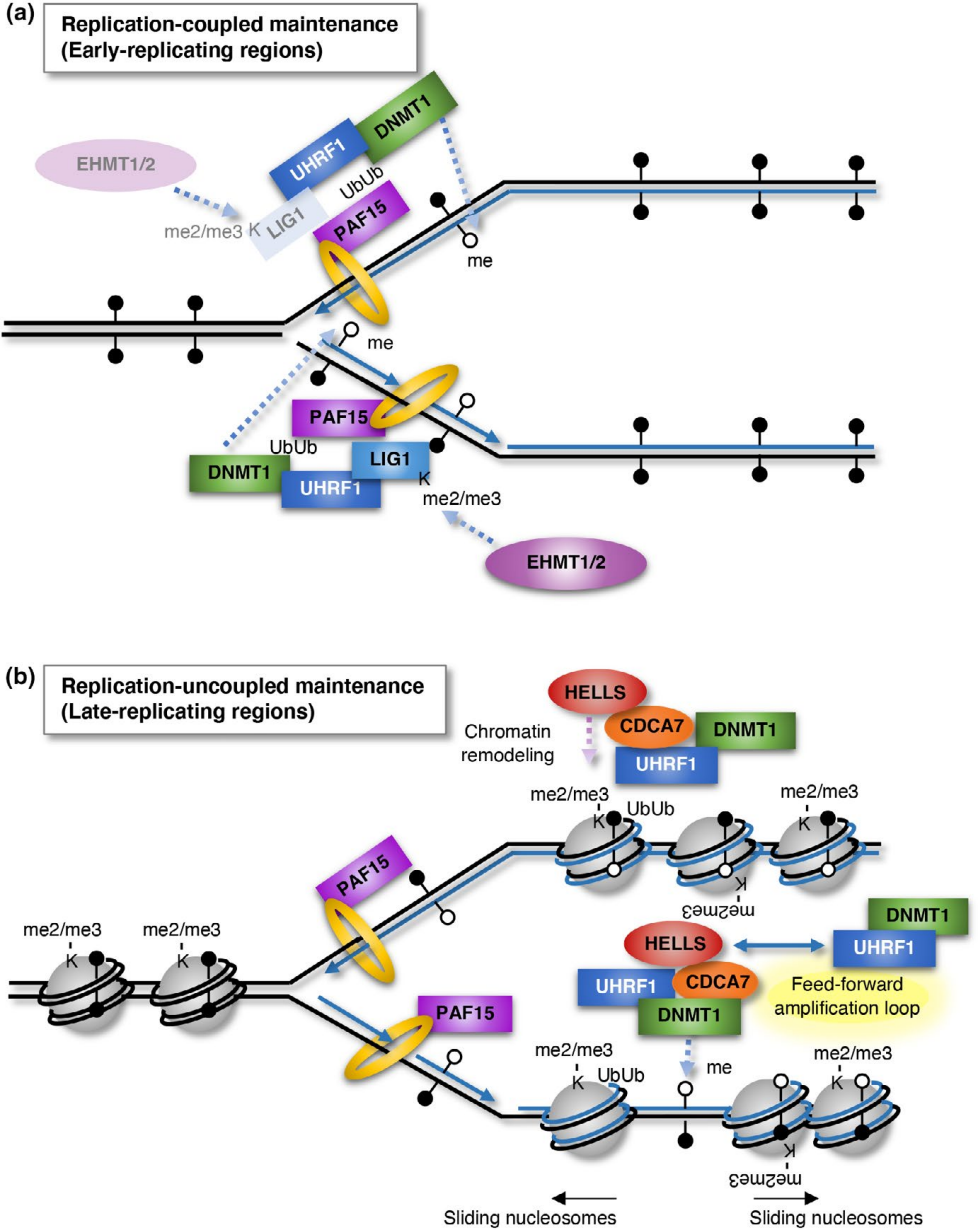
Models of replication‐coupled and replication‐uncoupled maintenance DNA methylation. (a) DNA methylation in early‐replicating regions is mostly maintained in a replication‐coupled manner. First, UHRF1 recognizes hemi‐methylated DNA through its SRA domain and mono‐ubiquitylates two histone lysine residues of PAF15 through its RING domain. Subsequently, DNMT1 recognizes the ubiquitylated PAF15 through its RFTS domain and methylates the unmethylated lysine residues in hemi‐methylated DNA. Both PAF15 and DNMT1 interact with PCNA through their PIP‐box, and the interactions likely support the simultaneous DNA replication and methylation maintenance. In addition to the above process, EHMT2 and EHMT1 methylate LIG1, which is critical for the joining of Okazaki fragments during lagging strand synthesis but can be required for maintenance DNA methylation of both DNA strands. Then, UHRF1 recognizes the methylated LIG1 through its TTD, and this interaction facilitates the recruitment of UHRF1 to DNA replicating sites. (b) DNA methylation of heterochromatic and late‐replicating regions is mostly maintained in a replication‐uncoupled manner. First, UHRF1 recognizes hemi‐methylated DNA through its SRA domain and H3K9me2/me3 through its TTD. Subsequently, UHRF1 mono‐ubiquitylates multiple lysine residues of histone H3 in nucleosomes through its RING domain and interacts with the CDCA7/HELLS complex in a feed‐forward manner, to slide nucleosomes, and generate bare hemi‐methylated DNA. Then, DNMT1 recognizes the ubiquitylated lysine residues and methylates the unmethylated cytosine residues in hemi‐methylated DNA. DNA methylation maintained in the replication‐uncoupled manner is prone to be lost after many cell divisions, as in cancer cells and aging cells. Black circles, methylated cytosines; white circles, unmethylated cytosines; yellow ring, PCNA

The core reaction of maintenance DNA methylation starts with the recognition of hemi‐methylated DNA by UHRF1 and the subsequent recruitment of DNMT1 to the site, to methylate unmethylated cytosine residues in hemi‐methylated DNA. Intriguingly, recent studies have revealed that auxiliary proteins modify this process in replication‐coupled and replication‐uncoupled manners (Ming et al., 2020; Petryk et al., 2020). Nishiyama et al. revealed that UHRF1‐mediated dual mono‐ubiquitylation of PAF15, which interacts with PCNA through its PCNA‐interacting protein (PIP) box (Emanuele et al., 2011), is required for maintenance DNA methylation at early‐replicating regions, whereas that of histone H3 is required for maintenance DNA methylation at late‐replicating regions (Nishiyama et al., 2020). DNMT1 recognizes the dual mono‐ubiquitylation of these proteins through the replication foci targeting sequence (RFTS) domain, and methylates the unmethylated cytosine residues in hemi‐methylated DNA. Contemporaneously, Ming et al. developed a novel technique called Hammer‐seq, which enabled the measurement of the kinetics of maintenance, and revealed that maintenance DNA methylation occurs in either replication‐coupled or replication‐uncoupled manners (Ming et al., 2020). The replication‐coupled process is governed by the PCNA–DNMT1 interaction through the recognition of PCNA by the PIP box of DNMT1. In this manner, more than 50% of methylated CpGs are successfully maintained, accompanied by fork passage (Ming et al., 2020). The methylation of early‐replicating regions is largely maintained via this mechanisms; thus both the PCNA–DNMT1 interaction and the PCNA–PAF15–UHRF1 interaction are required to accomplish this process (Figure 1a). This maintenance likely occurs before the wrapping of hemi‐methylated DNA around nucleosomes. The UHRF1–LIG1 interaction is also involved in the replication‐coupled process of maintenance DNA methylation (Ferry et al., 2017; Ming et al., 2020). Although LIG1 is critical for the joining of Okazaki fragments during lagging strand synthesis (Levin et al., 2000), it is unclear whether the UHRF1‐LIG1 interaction is required for the maintenance DNA methylation of both strands or only the lagging strand (Figure 1a). According to the Ferry et al., euchromatic histone lysine methyltransferase 2 (EHMT2, also called G9a) and EHMT1 (also called GLP) di‐ or tri‐methylate K126 of LIG1. Then, UHRF1 recognizes LIG1K126me2/me3, and this interaction facilitates the recruitment of UHRF1 to DNA replication sites (Figure 1a). Since maintenance of DNA methylation is faster in the lagging strands compared with the leading strands (Ming et al., 2020), there could be different maintenance manners between the two strands. However, further analysis is required for revealing which factor contributes to yield the difference.

In contrast, the replication‐uncoupled process is associated with H3K9me2/me3 (Ming et al., 2020), which are abundant modifications at heterochromatin corresponding to late‐replicating regions (Hiratani & Takahashi, 2019). At these regions, methylation by DNMT1 likely occurs after the wrapping of hemi‐methylated DNA around nucleosomes, because this type of maintenance exhibits much slower kinetics (up to 24 hr) than does replication‐coupled maintenance (Ming et al., 2020) and requires multiple mono‐ubiquitylation of histone H3 in nucleosomes by the RING domain of UHRF1 (Nishiyama et al., 2020), which stimulates Dnmt1 activity cooperating with the SRA domain of UHRF1 (Mishima et al., 2020). As chromatin acts as a barrier to DNA methylation, possibly because hemi‐methylated DNA wrapped around nucleosomes is not a suitable substrate for DNMT1 (Ming et al., 2020; Mishima et al., 2017), chromatin remodeling was expected to be required for replication‐uncoupled maintenance DNA methylation (Unoki, 2019). As expected, it was revealed that HELLS, which forms a chromatin remodeling complex with CDCA7 (Jenness et al., 2018), facilitates this type of maintenance by enhancing the chromatin association of UHRF1 (Figure 1b) (Han et al., 2020; Ming et al., 2020). According to Han et al., UHRF1 also facilitates the recruitment of HELLS to replication forks, resulting in a feed‐forward amplification loop between UHRF1 and HELLS (Han et al., 2020). Notably, the novel technique developed by Ming et al. answered the previously raised question of why DNA methylation in *LIG1*‐null cells is maintained normally (Ferry et al., 2017). Those authors showed that replication‐uncoupled maintenance DNA methylation is sufficient as a backup system of replication‐coupled maintenance (Ming et al., 2020).

Using the same method, Ming et al. also revealed that regions where DNA methylation loss occurs with aging are largely overlapped with the regions where DNA methylation is maintained in a replication‐uncoupled manner (Ming et al., 2020). It has been reported that DNA hypomethylation occurs with aging primarily in partially methylated domains (PMDs), which correspond to heterochromatic and late‐replicating regions (Zhou et al., 2018). It has also been reported that PMDs are hypomethylated in cancer cells and long‐cultured cells (Timp et al., 2014; Weber et al., 2005). Ming et al. showed that the CpGs with methylation loss during aging are clearly associated with the slowest maintenance kinetics (Ming et al., 2020), suggesting that replication‐uncoupled maintenance DNA methylation is prone to mistakes more than is the replication‐coupled maintenance process. It is easy to speculate that, in addition to the observation that replication‐uncoupled maintenance does not have a backup system (unlike replication‐coupled maintenance), the elimination of the chromatin barrier for methylation by DNMT1, which is required for replication‐uncoupled maintenance DNA methylation, is troublesome and could overlook some hemi‐methylated CpGs during the fast and limitless proliferation of cancer cells, as well as in aging cells, which have undergone repeated cell divisions through the lifetime span.

## 3 SYMPTOMS AND CAUSATIVE GENES OF ICF SYNDROME

**FIGURE 2:**
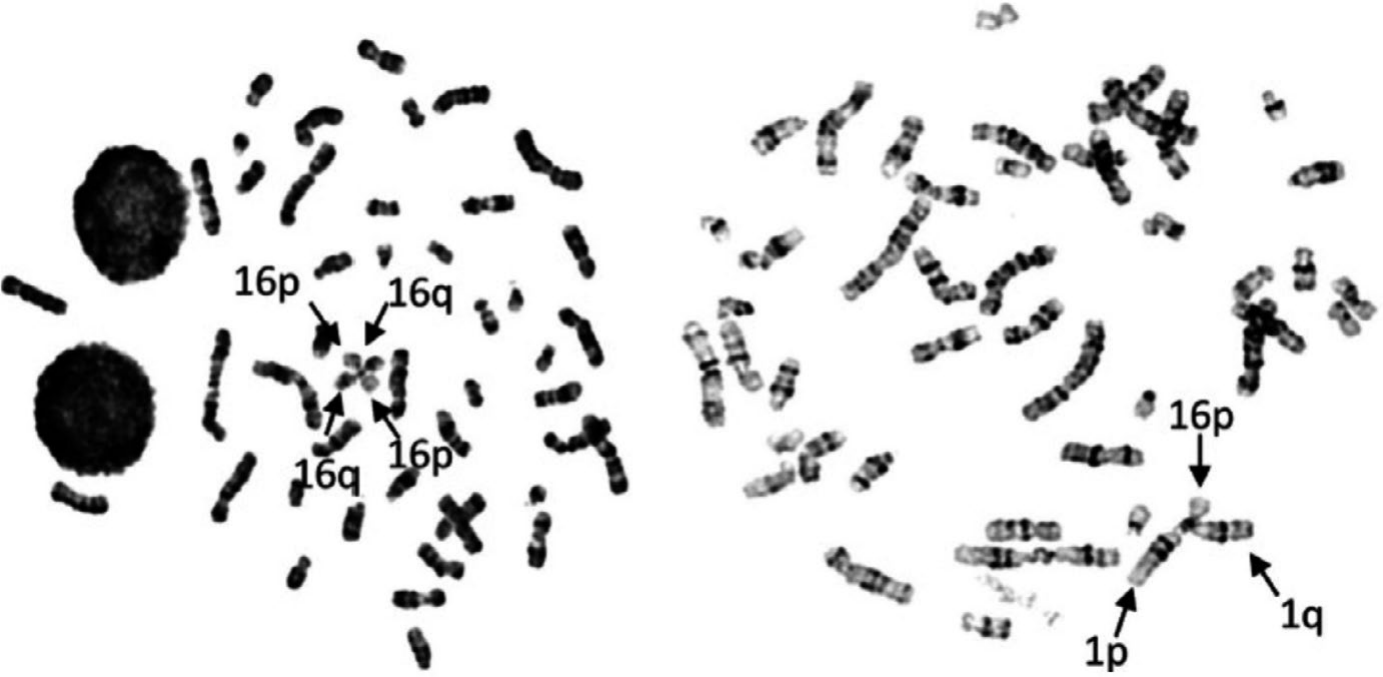
Representative multiradial chromosomes observed in the activated lymphocytes of a patient with ICF2 (Nitta et al., 2013). Multiradial configurations with multiple p and q arms derived from chromosomes 1 and 16, which have long pericentromeric satellite‐2 repeats, were observed (arrows)

ICF syndrome is a rare autosomal recessive congenital disease, the main symptom of which is reduced immunoglobulin levels in the serum resulting in recurrent infections (Ehrlich et al., 2006). Other variable symptoms of the syndrome include mild facial dysmorphism, failure to thrive, and psychomotor retardation (Ehrlich et al., 2006). The activated lymphocytes of these patients show centromeric/pericentromeric instability, which manifests as stretched heterochromatin, chromosome breaks, and multiradial chromosomes fused via the pericentromeric regions of chromosomes 1, 9, and 16 at high frequency (Figure 2) (Maraschio et al., 1988; Nitta et al., 2013). These cytological defects are accompanied by DNA hypomethylation in pericentromeric satellite‐2 and satellite‐3 repeats, which are especially abundant in these chromosomes (Vourc'h & Biamonti, 2011).

**FIGURE 3:**
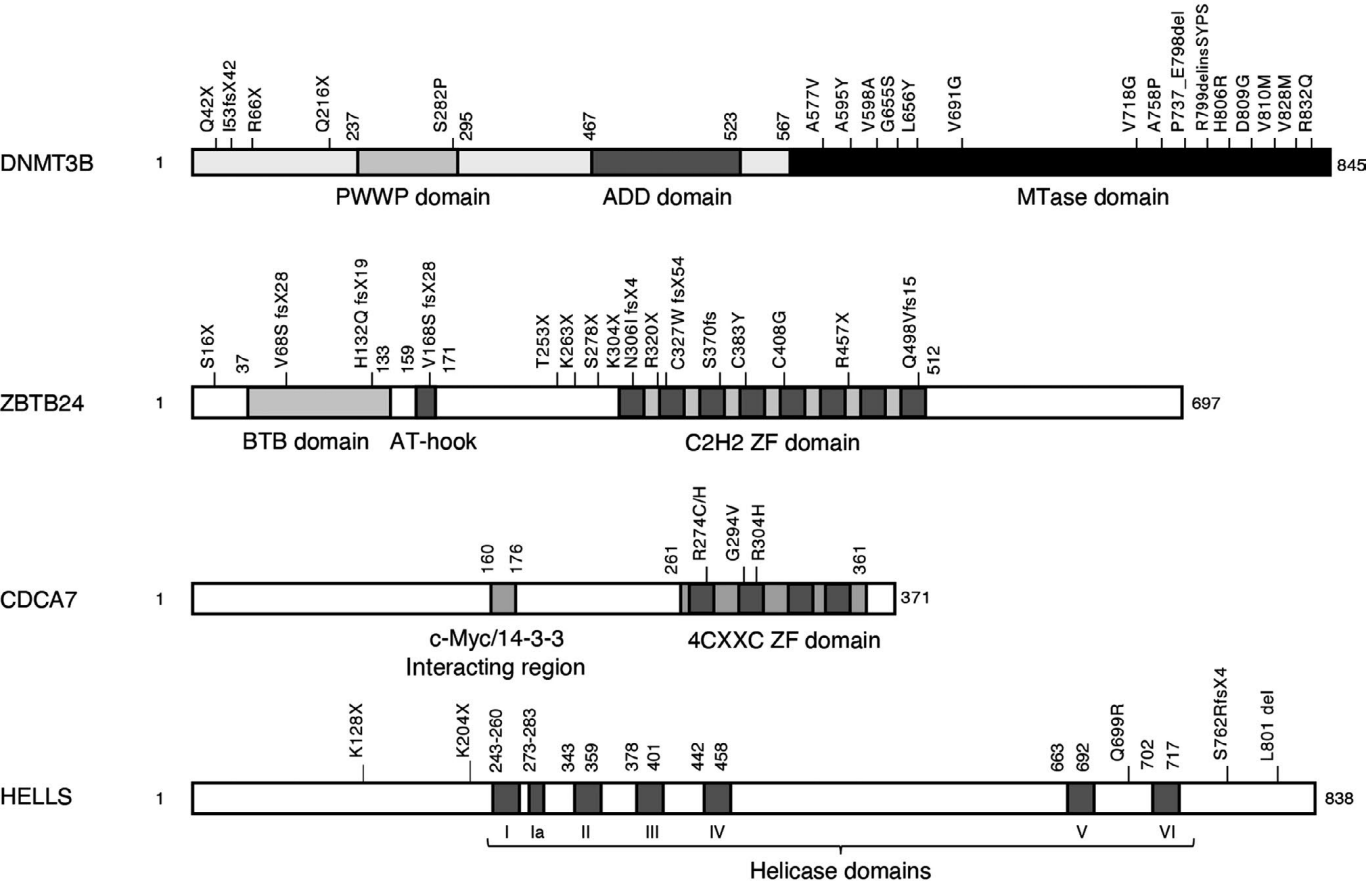
Protein structure of human DNMT3B, ZBTB24, CDCA7, and HELLS, and mutations found in patients with ICF. fs, frameshift; X, stop codon; PWWP, Pro‐Trp‐Trp‐Pro; ADD, ATRX‐DNMT3‐DNMT3L; BTB, BR‐C, ttk, and bab; ZF, zinc finger; I–VI, seven helicase domains (Jarvis et al., 1996)

Based on the causative genes identified to date, ICF syndrome is currently categorized into five subtypes: ICF1 (OMIM#242860), ICF2 (OMIM#614069), ICF3 (OMIM#616910), ICF4 (OMIM#616911), and ICFX (OMIM# is unavailable). Approximately 50% of patients with ICF possess mutations in the *DNMT3B* gene and are categorized as ICF1 (Hansen et al., 1999; Okano et al., 1999; Xu et al., 1999). In turn, approximately 30%, 10%, and 10% of patients with ICF possess mutations in the *zinc finger and BTB domain containing 24* (*ZBTB24*), *CDCA7*, and *HELLS* genes and are categorized as ICF2, ICF3, and ICF4, respectively (Table 1) (de Greef et al., 2011; Thijssen et al., 2015). The structures of the proteins encoded by these genes, and the mutations identified in the patients are summarized in Figure 3. The few patients, in whom causative gene(s) remain unknown, are tentatively categorized as ICFX; Recently, it is reported that the DNA methylation profile of two patients with ICFX resembles that of patients with ICF1, suggesting that at least a part of patients with ICFX may have abnormalities in regulatory regions of *DNMT3B* (Velasco et al., 2021). *ZBTB24* encodes a zinc finger protein that transcriptionally activates *CDCA7* (Wu et al., 2016). As described above, proteins encoded by *CDCA7* and *HELLS* constitute a chromatin remodeling complex possessing nucleosome‐sliding activity (Jenness et al., 2018). We revealed that this complex facilitates the nonhomologous end joining (NHEJ) of double‐strand breaks (DSBs) by enhancing access of Ku80 (also called XRCC5), a factor essential for NHEJ by protecting DSB ends from resection, to the sites of DSBs, and potentially suppresses many cytological defects; *ZBTB24* KO, *CDCA7* KO, and *HELLS* KO human embryonic kidney 293 (HEK293) cells generated using the CRISPR/Cas9 system displayed enlarged nuclei, centrosome amplification, abnormal chromosome segregation, and many related phenotypes, including proliferation defects, aneuploidy, and apoptosis (Unoki et al., 2019). Recently, it was reported that ZBTB24 and HELLS are essential for NHEJ during immunoglobulin class‐switch recombination (He et al., 2020; Helfricht et al., 2020). Taken together, these findings indicate that ZBTB24, CDCA7, and HELLS are largely involved in the same biological pathway, although they also have their specific functions; for example, ZBTB24 works not only as a transcriptional activator of *CDCA7*, but also as a direct regulator of poly(ADP‐ribose) polymerase 1 (PARP1)‐dependent NHEJ and class‐switch recombination (Helfricht et al., 2020).

**TABLE 1:** Causative genes and frequency of ICF syndrome

Types	ICF1	ICF2	ICF3	ICF4	ICFX
Causative genes	*DNMT3B*	*ZBTB24*	*CDCA7*	*HELLS*	Unknown
Frequency (%)	≈50%	≈30%	≈10%	≈10%	Few %

## 4 CHROMATIN REMODELING IN REPLICATION‐UNCOUPLED MAINTENANCE DNA METHYLATION

Region‐specific DNA methylation analyses have revealed the presence of DNA hypomethylation in pericentromeric satellite‐2 and satellite‐3 repeats and subtelomeres in ICF1 cells (Table 2) (Jiang et al., 2005; Sagie et al., 2017; Toubiana et al., 2018). On the other hand, DNA hypomethylation is observed in centromeric α‐satellite repeats in addition to pericentromeric repeats, but not in subtelomeres, in ICF2, ICF3, and ICF4 cells (Jiang et al., 2005; Toubiana et al., 2018) (Table 2). In addition to the rough features, a recent genome‐wide DNA methylation analysis using the Illumina Infinium HumanMethylation450 BeadChip (HM450K) revealed that the detailed genome‐wide DNA methylation patterns are also different between patients with ICF1 and those with other types of ICF syndrome; CpG islands are preferentially hypomethylated in the former, whereas CpG poor regions with heterochromatic and late‐replicating signatures are hypomethylated in patients with ICF2, ICF3, and ICF4 (Table 2) (Velasco et al., 2018). As described above, these regions correspond to the regions in which DNA methylation is maintained in a replication‐uncoupled manner (Figure 1b).

**TABLE 2:** DNA methylation status of patients with ICF syndrome

Types	ICF1	ICF2	ICF3	ICF4	References
Centromeric α‐satellite repeats	Normal	Hypo	Hypo	Hypo	Jiang et al., 2005
Pericentromeric satellite–2/3 repeats	Hypo	Hypo	Hypo	Hypo	Jiang et al., 2005
Subtelomeres	Hypo	Normal	Normal	Normal	Toubiana et al., 2018
Heterochromatic and late‐replicating regions (tendency)	Less affected	Hypo	Hypo	Hypo	Velasco et al., 2018

To examine whether DNMT3B, ZBTB24, CDCA7, and HELLS are involved in de novo or maintenance DNA methylation, the expression of these proteins was knocked down by siRNAs in mouse embryonic fibroblasts. The result revealed that knockdown of *Dnmt3b* did not affect the methylation status of centromeric minor satellite repeats, whereas knockdown of *Zbtb24*, *Cdca7,* and *Hells* reduced the DNA methylation levels of the repeats (Thijssen et al., 2015). A similar result was obtained using *DNMT3B* KO, *ZBTB24* KO, *CDCA7* KO, and *HELLS* KO HEK293 cells. Although DNA methylation at pericentromeric repeats was not significantly reduced in *DNMT3B* KO cells 2‐months after the KO procedure, it was almost completely diminished in *ZBTB24* KO, *CDCA7* KO, and *HELLS* KO cells (Unoki et al., 2019). Further, reintroduction of wild‐type proteins in these KO cells did not rescue the hypomethylation phenotype (Unoki et al., 2019). Taken together, these findings suggest that DNA hypomethylation by *DNMT3B* mutations in ICF1 cells is likely a result of a defect in de novo DNA methylation during the establishment of DNA methylation patterns in embryos, whereas that mediated by *ZBTB24*, *CDCA7*, and *HELLS* mutations in ICF2, ICF3, and ICF4 cells is likely a result of a defect in maintenance DNA methylation after the establishment of DNA methylation patterns.

To investigate further the functions of the CDCA7/HELLS complex, first we determined a number of CDCA7‐interacting proteins by immunoprecipitation and subsequent tandem mass spectrometric (IP‐MS/MS) analysis, and found that UHRF1 was involved in the CDCA7 interactors (Unoki et al., 2019). Later, it was also revealed that HELLS interacts with UHRF1 (Han et al., 2020), indicating that the CDCA7/HELLS complex tightly interacts with UHRF1. Second, we identified the proteins that are less accumulated on newly synthesized DNA strands in the absence of CDCA7 by isolation of proteins on nascent DNA (iPOND)‐MS/MS analysis using asynchronized wild‐type and *CDCA7* KO HEK293 cells. We found that the accumulation of DNMT1 and UHRF1 on newly synthesized strands was reduced by approximately 50% and 40%, respectively, in *CDCA7* KO cells (Unoki et al., 2020), suggesting that the CDCA7/HELLS complex is required for access of the DNMT1/UHRF1 complex to approximately half of newly synthesized strands. Considering that the CDCA7/HELLS complex is plausibly required for replication‐uncoupled maintenance DNA methylation (Figure 1b), this result is reasonable. The finding that the CDCA7/HELLS complex interacts with nucleosomes wrapped with unmethylated DNA in a sequence‐non‐specific manner in vitro (Jenness et al., 2018) indicates that, first, UHRF1 recruits the CDCA7/HELLS complex to the regions with hemi‐methylated DNA and H3K9me2/me3, where chromatin remodeling is required for replication‐uncoupled maintenance DNA methylation (Figure 1b). Then HELLS (probably together with CDCA7) further promotes the chromatin accessibility of UHRF1, and facilitates the maintenance DNA methylation by DNMT1 (Han et al., 2020; Ming et al., 2020), resulting in the feed‐forward amplification loop between UHRF1 and HELLS (Han et al., 2020). Hence, finally, all ICF‐related proteins fit in the right places of maintenance DNA methylation.

## 5 DNA METHYLATION AND CHROMOSOME STABILITY

Multiradial chromosomes fused via pericentromeric regions are observed in the activated lymphocytes of patients with all types of ICF. Although we revealed that the CDCA7/HELLS complex promotes the accumulation of Ku80 at DSBs and facilitates NHEJ repair (Unoki et al., 2019), no functions of DNMT3B in NHEJ have been reported. In addition, DNA hypomethylation at pericentromeric regions was not observed in *Ku80* hypomorphic mutant HEK293 cells (Unoki et al., 2019), and multiradial chromosomes have not been observed to date in *Ku80*‐null Chinese hamster and mouse cells, to the best of my knowledge. Therefore, a defect in NHEJ repair seems not to be a primary cause of these abnormal chromosomes; however, the DNA hypomethylation at pericentromeric regions, which are a common feature observed in all types of ICF syndrome, could be the primary cause of these chromosomes.

Our recent IP‐MS/MS and iPOND‐MS/MS analyses (described above) revealed that DExD‐Box Helicase 21 (DDX21) and SPT16 homolog facilitates chromatin remodeling subunit (SUPT16H), the latter of which forms the FACT heterodimeric chaperone with structure‐specific recognition protein 1 (SSRP1), interact with CDCA7, and are less accumulated on newly synthesized DNA strands in the absence of CDCA7 (Unoki et al., 2019, 2020). As DDX21 is involved in R‐loop resolution, and the FACT complex prevents R‐loop formation by organizing chromatin structure (Herrera‐Moyano et al., 2014; Song et al., 2017), we hypothesized that these proteins recruit the CDCA7/HELLS complex to near R‐loops in a feed‐forward amplification manner. An R‐loop is a structure composed of DNA:RNA hybrid and single‐stranded DNA that is generated physiologically during processes such as mitochondrial DNA replication and immunoglobulin class‐switch recombination and pathologically by failures in the resolution and/or prevention of ectopic R‐loops (Garcia‐Muse & Aguilera, 2019).

**FIGURE 4:**
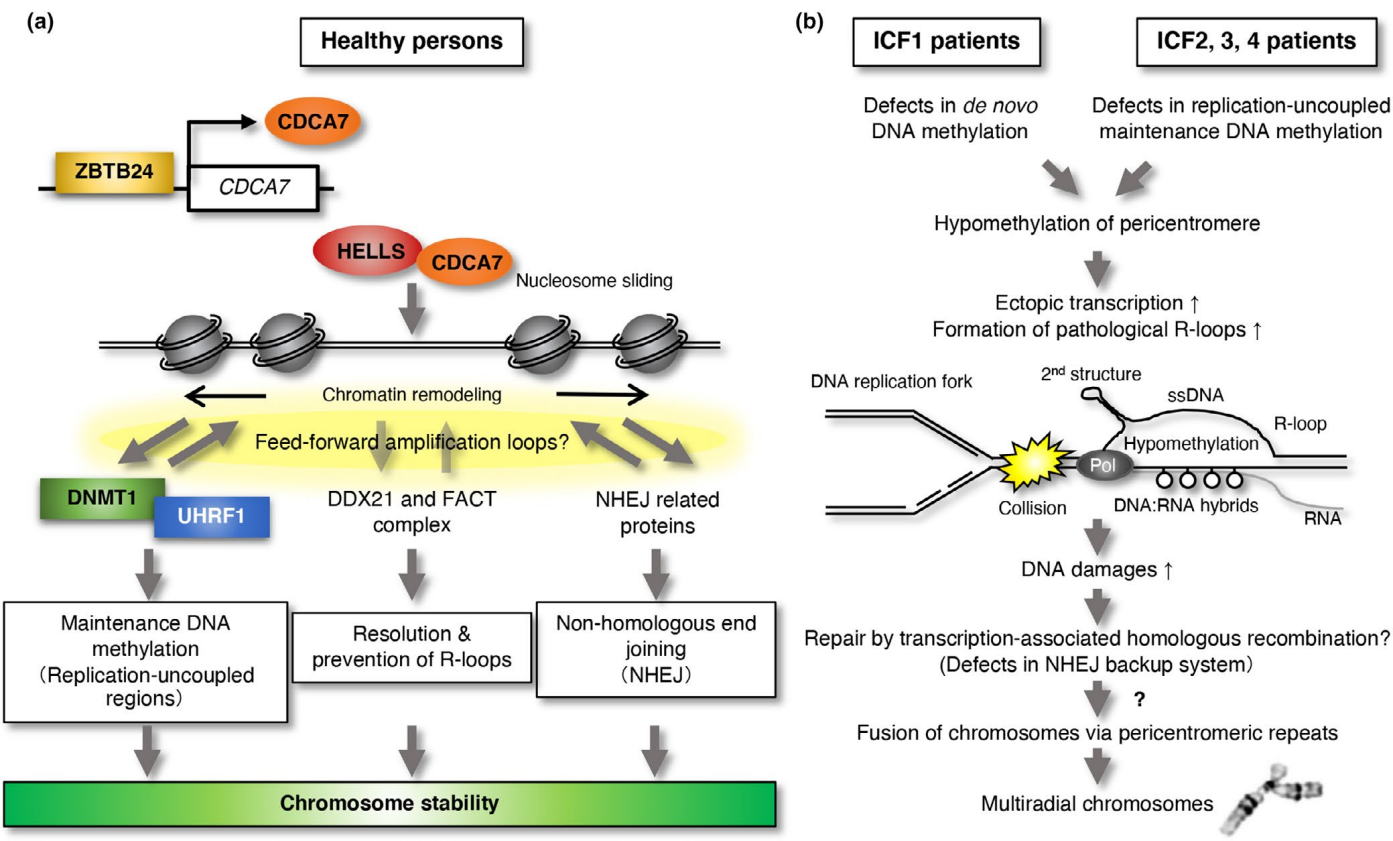
Potential mechanisms underlying the protection of chromosome stability in healthy persons and the molecular pathogenesis of ICF syndrome. (a) In healthy persons, the CDCA7/HELLS complex could promote the access of multiple proteins, including the DNMT1/UHRF1 complex, DDX21, the FACT complex, and NHEJ‐related proteins, to nucleosomes via chromatin remodeling activity, possibly in a feed‐forward manner. Through this activity, the complex likely maintains chromosome stability by facilitating the replication‐uncoupled maintenance DNA methylation of late‐replicating regions, including pericentromeric repeats (See Figure 1b), the resolution and/or prevention of R‐loops, and NHEJ. (b) In patients with ICF carrying mutations in *DNMT3B*, de novo DNA methylation is impaired, resulting in hypomethylation of a wide range of regions, including pericentromeric repeats. In patients with ICF2, ICF3, and ICF4 carrying mutations in *ZBTB24*, *CDCA7,* and *HELLS*, respectively, replication‐uncoupled maintenance DNA methylation is likely impaired, resulting in hypomethylation of late‐replicating regions including pericentromeric repeats. Subsequently, ectopic transcription from the regions occurs and pathological R‐loops are formed. This can cause DNA damage, possibly by collisions between the R‐loops and DNA replication forks. As DNA damage associated with R‐loop formation is preferentially repaired by transcription‐associated HR, and some ICF types have defects in NHEJ as a backup system of HR, the centromeric instability represented by multiradial chromosomes in ICF cells (see Figure 2) could result from unresolved Holliday junctions generated by aberrant HR between pericentromeric repeats in different chromosomes

In the nematode *Caenorhabditis elegans,* which does not possess DNA (CpG) methylation, heterochromatin comprising repetitive sequences is associated with H3K9 methylation, and the disruption of heterochromatin by the introduction of mutations in its two H3K9 methyltransferases, *met‐2* and *set‐25*, triggers aberrant transcription from the sequences, which results in DNA:RNA hybrid‐associated repeat instability (Zeller et al., 2016). Therefore, we asked whether the DNA hypomethylation of pericentromeric repeats in *ZBTB24* KO, *CDCA7* KO, and *HELLS* KO cells causes chromosome instability via similar process. Consistent with the hypomethylation state of the repeats, the transcription and formation of aberrant DNA:RNA hybrids at the repeats were significantly increased in *ZBTB24* KO, *CDCA7* KO, and *HELLS* KO cells (Unoki et al., 2019, 2020). Since it is reported that DNA:RNA hybrids and R‐loops are poor substrates for DNMT1 (Ross et al., 2010), this may accelerate loss of methylation at the repeats in these KO cells. Furthermore, ectopic expression of RNASEH1, which digests DNA:RNA hybrids and resolves R‐loops, reduced the accumulation of DSBs at a broad range of genomic regions, including satellite‐2 repeats, in these cells. Hence, we propose that hypomethylation resulting from inefficient DNMT1/UHRF1 recruitment at pericentromeric repeats by defects in the CDCA7/HELLS complex induces aberrant transcription from the repeats, which results in pathological R‐loop formation and subsequent DSBs, possibly via collisions of the R‐loops and DNA replication forks, etc (Garcia‐Muse & Aguilera, 2019). As DNA damages associated with R‐loops are preferentially repaired by transcription‐associated HR (Yasuhara et al., 2018), the centromeric instability represented by multiradial chromosomes observed in ICF cells may be the result of unresolved Holliday junctions generated by incorrect strand exchanges during HR between highly conserved pericentromeric repeats in different chromosomes (Maraschio et al., 1988; Tuck‐Muller et al., 2000). In *ZBTB24* KO, *CDCA7* KO, and *HELLS* KO cells, the defect in NHEJ could promote the usage of HR (Unoki et al., 2019). The possible mechanisms via which chromosome stability is protected in healthy persons and DNA hypomethylation causes the formation of multiradial chromosomes in patients with ICF syndrome are summarized in Figure 4a,4b.

## 6 CONCLUDING REMARKS

After the identification of *CDCA7* and *HELLS* in 2015 as causative genes of ICF syndrome (Thijssen et al., 2015), the outline of the molecular pathogenesis regarding the chromosome instability observed in this syndrome has been largely unveiled, although further detailed studies are required. Our recent studies of ICF‐related proteins have deepened our insights into the molecular mechanisms of maintenance DNA methylation and chromosome stability (Unoki et al., 2019, 2020). The recent studies showing that ZBTB24 and HELLS are involved in NHEJ during immunoglobulin class‐switch recombination (He et al., 2020; Helfricht et al., 2020) have also deepened our insights into the molecular mechanisms of antibody production. Despite its rareness, studies of ICF syndrome have revealed many scientifically important propositions and are surely expected to provide additional insights regarding maintenance DNA methylation, chromosome stability, antibody production, morphogenesis of the face, and neurogenesis during development.
